# Dopamine D3 receptor dysfunction prevents anti-nociceptive effects of morphine in the spinal cord

**DOI:** 10.3389/fncir.2014.00062

**Published:** 2014-06-11

**Authors:** Kori L. Brewer, Christine A. Baran, Brian R. Whitfield, A. Marley Jensen, Stefan Clemens

**Affiliations:** ^1^Department of Emergency Medicine, Brody School of Medicine, East Carolina UniversityGreenville, NC, USA; ^2^Department of Physiology, Brody School of Medicine, East Carolina UniversityGreenville, NC, USA

**Keywords:** dopamine d3 receptor, mu-opiod receptor, nociception, second messenger cross-talk, dopamine d1 recptor, spinal reflexes

## Abstract

Dopamine (DA) modulates spinal reflexes, including nociceptive reflexes, in part via the D3 receptor subtype. We have previously shown that mice lacking the functional D3 receptor (D3KO) exhibit decreased paw withdrawal latencies from painful thermal stimuli. Altering the DA system in the CNS, including D1 and D3 receptor systems, reduces the ability of opioids to provide analgesia. Here, we tested if the increased pain sensitivity in D3KO might result from a modified μ-opioid receptor (MOR) function at the spinal cord level. As D1 and D3 receptor subtypes have competing cellular effects and can form heterodimers, we tested if the changes in MOR function may be mediated in D3KO through the functionally intact D1 receptor system. We assessed thermal paw withdrawal latencies in D3KO and wild type (WT) mice before and after systemic treatment with morphine, determined MOR and phosphorylated MOR (p-MOR) protein expression levels in lumbar spinal cords, and tested the functional effects of DA and MOR receptor agonists in the isolated spinal cord. *In vivo*, a single morphine administration (2 mg/kg) increased withdrawal latencies in WT but not D3KO, and these differential effects were mimicked *in vitro*, where morphine modulated spinal reflex amplitudes (SRAs) in WT but not D3KO. Total MOR protein expression levels were similar between WT and D3KO, but the ratio of pMOR/total MOR was higher in D3KO. Blocking D3 receptors in the isolated WT cord precluded morphine's inhibitory effects observed under control conditions. Lastly, we observed an increase in D1 receptor protein expression in the lumbar spinal cord of D3KO. Our data suggest that the D3 receptor modulates the MOR system in the spinal cord, and that a dysfunction of the D3 receptor can induce a morphine-resistant state. We propose that the D3KO mouse may serve as a model to study the onset of morphine resistance at the spinal cord level, the primary processing site of the nociceptive pathway.

## Introduction

Opiate analgesics are the classical first line treatment for strong and persistent pain, but their effectiveness in long-term treatment is limited by the emergence of tolerance (Colpaert, 2002; Dupen et al., 2007; Bekhit, 2010; Joseph et al., 2010). This morphine-resistant condition is thought to arise over time and involve a dysfunction of μ-opioid receptor (MOR)- and dopamine (DA)-receptor mediated cAMP/PKA second messenger pathways in the brain (Suzuki et al., 2001; Schmidt et al., 2002; Fazli-Tabaei et al., 2006; Zhang et al., 2008, 2012; Le Marec et al., 2011; Enoksson et al., 2012). However, under this tenet the role of the spinal cord remains overlooked. The spinal cord is the first site for the processing for nociceptive information, and it houses both DA and MOR receptors in the dorsal horn (Abbadie et al., 2001; Levant and McCarson, 2001; Ray and Wadhwa, 2004; Zhu et al., 2007, 2008). DA regulates spinal cord circuits, including pain-associated responses (Garraway and Hochman, 2001; Clemens and Hochman, 2004; Yang et al., 2005; Keeler et al., 2012), and both D1 and D3 receptors are present in the dorsal horn (Levant and McCarson, 2001; Zhu et al., 2007). Further, the MOR is associated with inhibitory G proteins (G*_i_* and G*_o_*) and is present in the spinal cord (Mansour et al., 1994; Ji et al., 1995; Ray and Wadhwa, 1999, 2004; Abbadie et al., 2001, 2002; Zhang et al., 2006). Upon ligand binding to the receptor, signaling cascades are activated that inhibit cAMP production, resulting in the opening of K^+^ channels or closing of Ca^2+^ channels (Connor and Christie, 1999; Connor et al., 1999; Williams et al., 2001). Its desensitization is under the control of G-protein-coupled receptor kinase (GRK)-mediated phosphorylation (Connor et al., 2004; Gainetdinov et al., 2004; Garzon et al., 2005), followed by ß-arrestin-mediated internalization (Bohn et al., 1999, 2000; Ohsawa et al., 2003; Connor et al., 2004). Blocking ß-arrestin expression improves morphine-mediated analgesia (Li et al., 2009), and recent data suggest that morphine-induced ß-arrestin complex formation primarily requires D1 receptors (Urs et al., 2011).

Interestingly, D1 receptors form heterodimers with D3 receptors (Surmeier et al., 1996; Fiorentini et al., 2008; Maggio et al., 2009; Missale et al., 2010; Cruz-Trujillo et al., 2013) and both play a role in opioid tolerance (Lin et al., 1996; Cook et al., 2000; Richtand et al., 2000; Fazli-Tabaei et al., 2006). We recently found that animals lacking a functional D3 receptor (D3KO) exhibited facilitated pain-associated reflexes (Keeler et al., 2012), and we address here the role of the spinal D3 receptor on MOR functional states and morphine responsiveness.

We evaluated thermal pain paw withdrawal latencies in D3KO and wild type (WT) controls before and after administration of morphine, determined naïve MOR and phosphorylated MOR (p-MOR) protein expression levels in the lumbar spinal cord, and compared spinal reflex amplitudes (SRA) in isolated spinal cords from WT and D3KO *in vitro*, to assess the role of the D3 receptor in mediating morphine actions in isolated WT spinal cords.

We found that naïve D3KO were unresponsive to morphine administration, both *in vivo* and *in vitro*, and that this lack of responsiveness was associated with increased levels of spinal pMOR in D3KO. Moreover, while the highly-specific MOR agonist, DAMGO, was able to decrease SRAs in both WT and D3KO *in vitro*, this effect was significantly smaller in D3KO than WT. Finally, acute pharmacological block of the D3 receptor in the isolated WT cord was sufficient to induce a D3KO-like phenotype and prevent the modulatory actions of morphine on SRAs observed under control conditions.

## Materials and methods

All experimental procedures complied with NIH guidelines for animal care and were approved by the East Carolina University Institutional Animal Care and Use Committee. Male dopamine D3 receptor knockout mice (D3KO; strain B6.129S4-*Drd3^tm^*1*^dac^/J;* stock # 002958, Jackson Laboratory, Bar Harbor, ME) and their appropriate associated wild-type (WT) controls (C57BL/6) were used for *in vivo* behavioral testing and Western blot experiments (3–4 months), while neonatal pups of either sex were used for extracellular electrophysiology and pharmacology (postnatal days 7–14).

### Behavioral assessments

Thermal withdrawal latencies were tested on 10 D3KO and 8 WT males using a Hargreaves apparatus. Prior to baseline testing, mice were acclimated to the apparatus by being placed in the apparatus for 2 h per day for a total of 5 days. Baseline thresholds were obtained over three test sessions, with each test session occurring at the same time of day, and each mouse being placed in the chamber to which it was acclimated. Each test session consisted of three trials (i.e., application of the heat stimulus at 56°C) separated by at least 5 min. The latencies obtained over the 3 trials were averaged to get the mean baseline latency for each test day. After baselines were established, a randomly chosen subset of mice (5 D3KO and 4 WT) was administered morphine sulfate (Sigma, 2 mg/kg, i.p.) and tested 30 min later as outlined above.

### Tissue collection and western blot analysis

One week after behavioral testing, animals were deeply anesthetized with inhaled isoflurane (4–5%), decapitated, and spinal cords were dissected, frozen in liquid nitrogen and stored at -80°C. Proteins were homogenized in RIPA buffer containing protease and phosphatase inhibitors (Sigma-Aldrich, St. Lois, MO), lysates centrifuged, and the supernatant collected. Total protein was quantified using an EZQ Quantitation Kit (R33200; Invitrogen, Grand Island, NY). Equal concentrations of protein were separated using a SDS-PAGE (Criterion TGX Any kD, Bio-Rad, Canton, MA) and transferred onto a PVDF membrane (Immobolin-P, Millipore, Germany). To verify consistent protein transfer across the lanes, we measured total protein staining of the membrane with Coomassie Blue, and compared the protein staining with the β-actin expression in the corresponding lanes. We did not observe any significant difference in β-actin protein expression between WT and D3KO (*p* = 0.54 and 0.2, respectively). For MOR and pMOR protein expression assessments, membranes were blocked using 5% BSA overnight and probed with primary antibodies for the MOR (ab10275, Abcam, UK) at 1:1000 and p-MOR (bs3724R, BIOSS, UK) at 1:1000 overnight. This MOR antibody can detect bands at ~50 kDa (Kerros et al., 2010) and has been verified in a different study by comparing it to the effects of another MOR antibody (Loyd et al., 2008). The pMOR antibody we used shows a strong band at ~44 kDa (http://biossusa.com/store/bs-3724r.html), and control ELISA data provided by BIOSS show an increased binding of this antibody to pMOR over MOR (data not shown, BIOSS, personal communication). Membranes were washed and incubated in anti-mouse and anti-rabbit secondary antibodies (R&D Systems, Minneapolis, MN) respectively. Target proteins were visualized using ECL Plus detection reagent (80916; Invitrogen, Grand Island, NY) according to the manufacturer's recommendations, quantified using relative integrated density normalized against β-actin (ab8226, Abcam, UK) at 1:2000, and analyzed with ImageJ software (version 1.48S, NIH). Expression values are given as the ratio of MOR or pMOR to β-actin.

For D1 receptor protein assessments, membranes were blocked overnight using 5% BSA at 4°C and probed with primary antibodies for the D1 receptor (ab81296, Abcam, Cambridge, MA) and β-actin (ab8226, Abcam, UK) at 1:1000 overnight. This D1 receptor antibody can detect bands at both 50 and 75 kDa (Mizuta et al., 2013). Membranes were washed four times at 5 min in TBS-T and incubated for 30 min at room temperature using 5% BSA in anti-rabbit IR800 (35571, Thermo Scientific, Rockford, IL) and anti-mouse IR680 (926-68070, Li-Cor, Lincoln, NE) secondary antibodies at 1:30000. The membranes were then washed another three times for 5 min in TBS-T followed by two washes at 5 min in PBS. Target proteins were visualized using the Li-Cor detection system (Odyssey Clx, Li-Cor Biosciences, Lincoln, NE) and associated software (Image Studio, Li-Cor), and quantified with ImageJ. D1 receptor protein expression values were normalized to β-actin protein expression.

### Electrophysiology and pharmacology

A total of 19 WT and 17 D3KO neonatal pups of either sex (postnatal days 7–14) were used for extracellular electrophysiological and pharmacological experiments. As reported earlier (Clemens and Hochman, 2004; Keeler et al., 2012), animals were deeply anesthetized with i.p. injection (50 μl/10 g) of a ketamine (90 mg/ml)/xylazine (10 mg/ml) mix, and after verification of deep anesthesia decapitated. Spinal cords were removed quickly, usually completed within 10 min, placed in a Sylgard-lined Petri dish, the Dura mater desheathed and the cords hemisected. Throughout this process the preparations were submersed in oxygenated (95 O_2_/5% CO_2_) ice-cold high-sucrose artificial cerebrospinal fluid (containing in mM: 342 sucrose, 180.2 Glucose, 203.3 MgCl_2_, 147.02 CaCl_2_, 137.99 NaH_2_PO_4_, 84.01 NaHCO_3_, 74.56 KCl, pH 7.4). After these initial dissection steps, the high-sucrose solution replaced with oxygenated artificial cerebrospinal fluid (ACSF; containing in mM: 128 NaCl, 1.9 KCl, 10 Glucose, 1.3 MgSO_4_, 2.4 CaCl_2_, 1.2 KH_2_PO_4_, 26 NaHCO_3_, pH 7.4), acclimated to room temperature. Subsequently, small glass suction electrodes were carefully attached to identified dorsal and ventral lumbar roots (usually L2-L5).

After a recovery of phase of ~30–60 min, reflex responses were elicited with a constant current stimulator (Iso-Stim 01D, NPI Electronics, Tamm, Germany, or a custom-built Linear Isolation Unit, Model MI 401, Department of Animal Physiology, University of Cologne) with pulses of 100**–**500 μ A for 100**–**250 μs. Signals were recorded and amplified with a 4-channel differential AC amplifier (Model 1700, A-M Systems, Sequim, WA), digitized with a Digidata 1440A, and analyzed with pClamp v.10.2 software (Molecular Devices, Sunnyvale, CA). Single pulse stimulations were delivered every 60 s (WT) or 120**–**180 s (D3KO) to avoid habituation (data not shown). Reflex responses were recorded and analyzed by rectified integration of the recorded responses. SRAs were obtained before (control conditions) and during application of morphine sulfate (Sigma, 1 μM), the D3 receptor-preferring antagonist, nafadotride (Tocris, 20 μM), or the specific MOR agonist DAMGO (Tocris, 10 μM). For testing the interactions between the D3 and MOR in the WT spinal cord, we first bath-applied the D3-receptor preferring antagonist, nafadotride, to establish the effects of this drug, before adding morphine to the bath. Drugs were bath-applied and for 30**–**60 min, during which time the stimulus protocol was maintained. Following the drug application testing, drugs were carefully washed out (through ACSF exchange of 4–5 times the bath volume). For analytical purposes, we compared the last 10 consecutive SRAs in the control condition with the last 10 SRAs during the drug application.

### Statistical analysis

For behavioral experiments, baseline and post-morphine thermal thresholds were compared between WT and D3KO mice using a RM ANOVA (SigmaPlot, Systat, San Jose, CA) with *p* < 0.05 indicating significance. For determining differences in MOR and p-MOR protein expression levels between groups, we used using a paired *t*-test (JMP v.10, SAS, Inc., Cary NC) with *p* < 0.05 indicating significance. For electrophysiological experiments, SRAs under drug conditions were averaged and normalized to the mean of the pre-drug values, and statistical significance was determined with *t*-tests or ANOVA and subsequent *post-hoc* comparisons, as appropriate, and with α set <0.05 (SigmaPlot, Systat, San Jose, CA).

## Results

### Behavior

**Figure 1:**
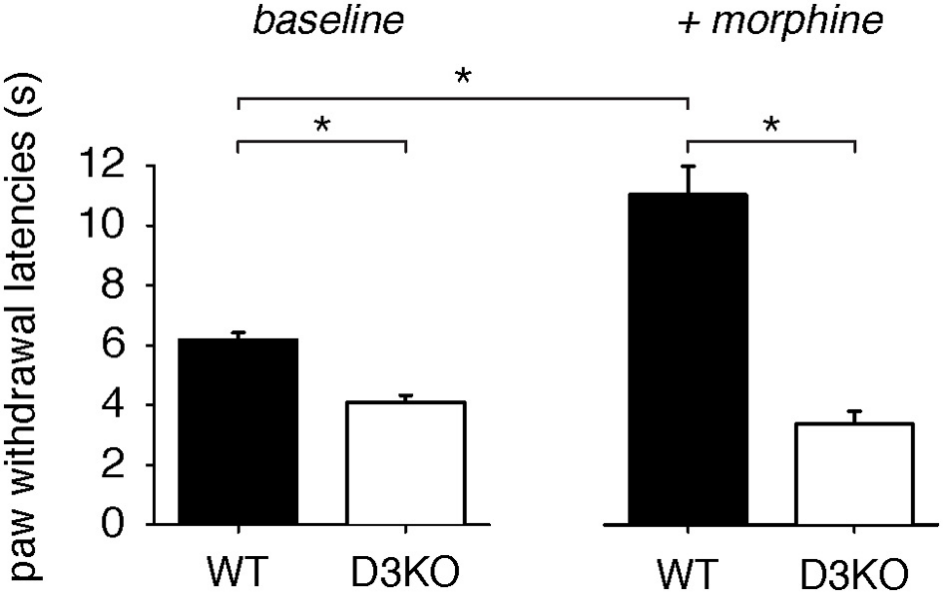
**Thermal pain withdrawal latencies at baseline and after a single i.p. application of 2 mg/kg morphine. Left panel:** Baseline data for WT (*n* = 8) and D3KO (*n* = 10). Under control conditions, D3KO displayed a significantly lower time to withdrawal than WT. **Right panel:** D3KO (*n* = 5) continued to maintain a lower withdrawal time than WT (*n* = 4), but morphine significantly increased latencies in WT but not D3KO. * indicates *p* < 0.001.

Under baseline conditions, pain withdrawal latencies of WT were significantly higher than those of D3KO (WT: 6.24 ± 0.64, *n* = 4; D3KO: 4.84 ± 1.08 s, *p* < 0.001, *n* = 5, *t*-test, Figure 1, left panel), suggesting an increased excitability to thermal stimulation of the hindpaw in the D3KO animals. A single injection of morphine (2 mg/kg, i.p.) significantly increased thermal withdrawal latencies in WT from 6.24 ± 0.64 s to 11.01 ± 1.91 s (*p* < 0.001, Figure 1, right panel), whereas they remained unaltered in D3KO (4.84 ± 1.08 s, vs. 3.94 ± 0.95, *p* = 0.902, RM-ANOVA). Consequently, the difference in pain withdrawal latencies between morphine-treated WT and D3KO remained significant (*p* < 0.001, *t*-test). These data suggest that under naïve conditions D3KO are unresponsive to the system-wide application of morphine.

### MOR protein expression in the lumbar cord

**Figure 2:**
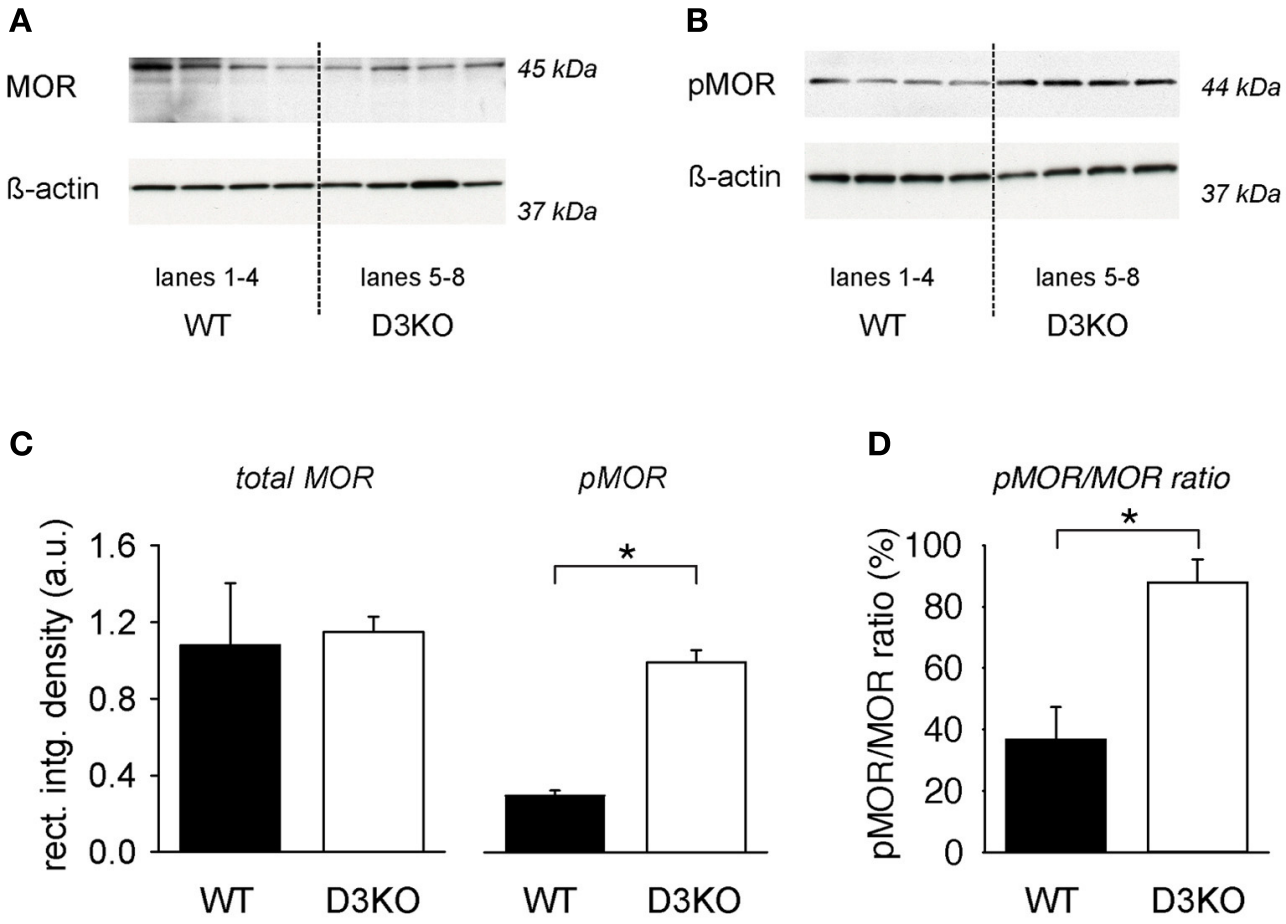
**Protein expression levels of total MOR and pMOR in lumbar spinal cord, normalized to β-actin. (A)** Total MOR protein expression in WT and D3KO (top panels), and their respective ß-actin protein expressions (bottom panels). Lanes 1–4 represent lumbar spinal cords from four independent WT, while lanes 5–8 represent tissues from four independent D3KO. Total MOR protein expression levels were overall similar between WT and D3KO. Note that we first probed for MOR expression before stripping and re-probing for ß-actin. **(B)** pMOR protein expression levels in WT and D3KO (top lanes), and their respective ß-actin protein expression (bottom panels). Note that pMOR expression levels showed a stronger labeling in D3KO than WT. **(C)** Quantification of expression data: After normalization of MOR and pMOR data to their respective ß-actin expression in each lane, total MOR protein expression was similar between WT and D3KO (^*^p = 0.85, *n* = 4 each). In contrast, pMOR protein expression was significantly increased in D3KO over WT (*p* < 0.001, *n* = 4 each). **(D)** Ratio of pMOR/MOR protein expression in the lumbar spinal cord of WT and D3KO. In WT, pMOR was low, whereas in D3KO a large majority of MORs was phosphorylated (^*^p = 0.008, *n* = 4 each).

To test if changes in MOR levels or phosphorylation status might underlie the lack of the opioid effect observed in D3KO, we assessed MOR and pMOR protein expression levels in lumbar spinal cords of WT and D3KO (Figure 2). We probed lumbar spinal cords of 4 naïve (untreated) WT and 4 D3KO for MOR (Figure 2A) and pMOR protein expression (Figure 2B). In both experiments, we first probed for the respective receptor protein expression before stripping the blot and then probing for ß-actin. We found that, after normalization to ß-actin, WT and D3KO had similar levels of total MOR (WT: 1.08 ± 0.32 a.u.; D3KO: 1.146 ± 0.08 a.u., *p* = 0.85, *n* = 4; Figure 2C, left panels). In contrast, pMOR protein expression levels of pMOR in D3KO were significantly elevated over WT (WT: 0.127 ± 0.03 a.u., D3KO: 0.992 ± 0.13 a.u., *p* < 0.001; Figure 2C, *p* < 0.01, right panels). As overall MOR but not pMOR expression levels were similar, and both sample groups passed Normality (Shapiro-Wilk; *p* = 0.441 and *p* = 0.509, respectively) and Equal Variance Tests (*p* = 0.085 and *p* = 0.505, respectively), we next calculated the ratio of pMOR to MOR expression levels (Figure 2D). We found that in WT 36.7 ± 10.6% of total MOR was phosphorylated, while in D3KO this rate 87.8 ± 7.6% (*p* = 0.008), suggesting a reduced availability of the MOR for ligand binding in D3KO.

### Extracellular electrophysiology and pharmacology

**Figure 3:**
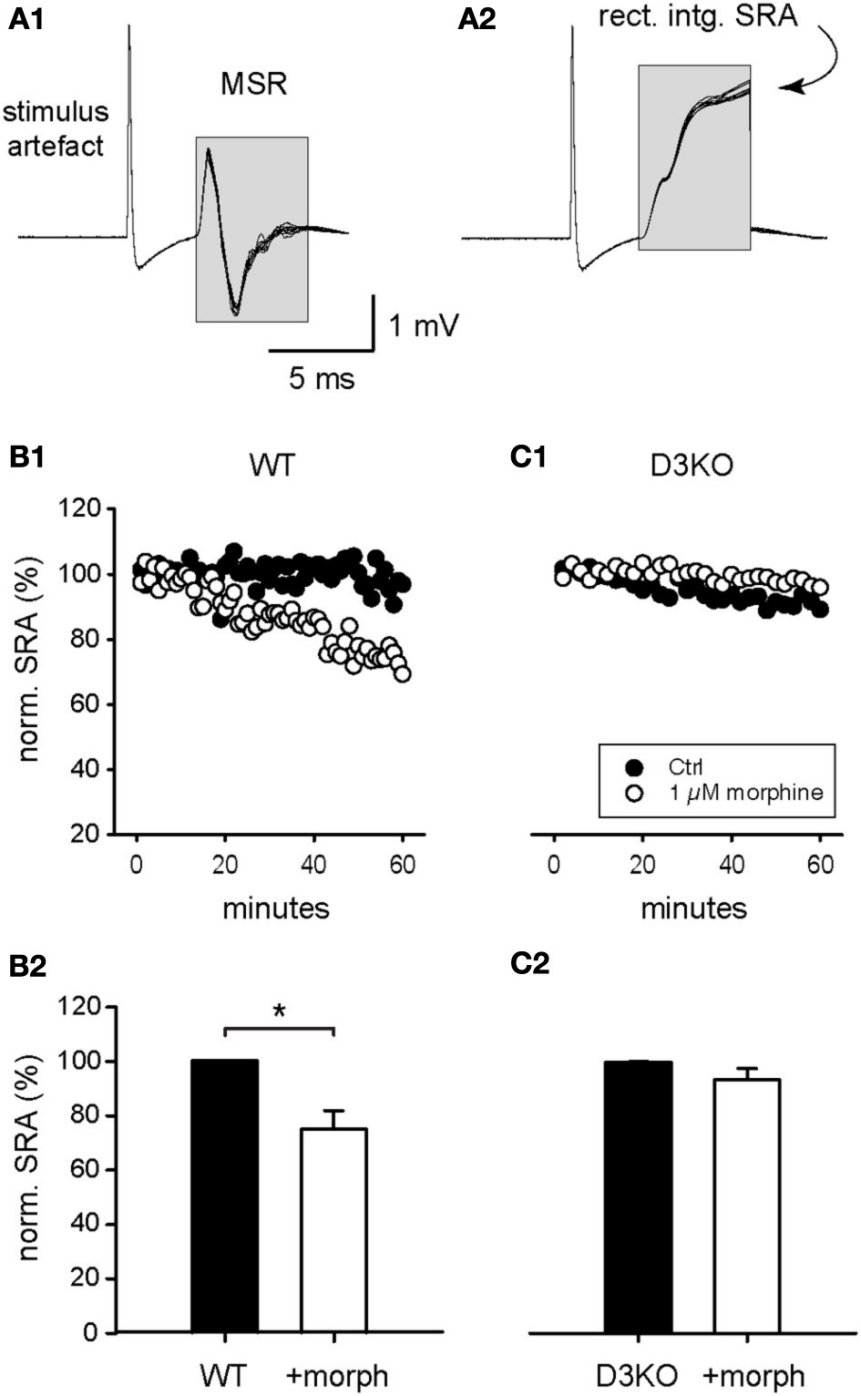
**Modulation of spinal reflex amplitudes (SRAs) by morphine. (A1)** Example of a reflex response recorded from ventral roots (10 consecutive sweeps superimposed). Stimulation of a dorsal root (at stimulus artifact) induced a monosynaptic reflex response (MSR) in the corresponding ventral root. The boxed region identifies the epochs chosen to calculate the amplitude of the spinal reflex amplitude (SRA) represented in **(A2)**. **(A2)** The rectification and subsequent integration of the epochs identified in **(A1)** led to the signal values highlighted in the boxed region. The final maximal values of each epoch were used as a measurement of the SRA for each sweep, and used in the subsequent analysis steps represented in panels **(B,C)**, and Figures 4, 5. **(B1)** Typical time course of control SRAs in WT before and during bath-application of 1 μM morphine (control: black symbols; morphine: white symbols). During morphine, reflex amplitudes gradually decreased in amplitude. **(B2)** Normalized SRAs in WT (*n* = 6). Morphine led to a significant reduction in SRA. ^*^p = 0.004. **(C1)** Typical time course of control SRAs in D3KO before and during bath-application of 1 μM morphine (control: black symbols; morphine: white symbols). **(C2)** Normalized SRAs in D3KO (*n* = 12). In these animals, morphine had no significant effect on SRA.

To test the functional effects of morphine mediated responses at the spinal cord level alone, we next elicited and recorded SRAs in isolated spinal cords of WT and D3KO before and during exogenous application of morphine (Figure 3). Constant-current stimulation of dorsal lumbar roots via glass suction electrodes elicited within a few milliseconds a monosynaptic stretch reflex on the corresponding ventral roots (MSR, **Figure 3A1**) that generally lasted less than 5–6 ms. The signal traces in the MSR time window were rectified and integrated, and the resulting data points at the end of each sweep were used as a measure of SRA (**Figure 3A2**).

Bath-application of morphine (1 μM) induced in WT a gradual decrease in SRA amplitude (**Figure 3B1**), and led to a significant reduction in WT SRAs at the end of the recorded application interval, from 100.3 ± 0.2 to 75.2 ± 6.8% (*p* = 0.004, *n* = 6, **Figure 3B2**). In contrast, in D3KO, bath-application of 1 μM morphine had no significant modulatory effect on SRA (**Figures 3C1,C2**; control: 99.8 ± 0.2%; morphine: 93.2 ± 4.2%, *p* = 0.052, *n* = 12). These data mirror the effects observed in the thermal pain paradigm, and they suggest that the behavioral changes observed in the intact animals stem from changes in the D3 receptor system at the spinal cord level.

**Figure 4:**
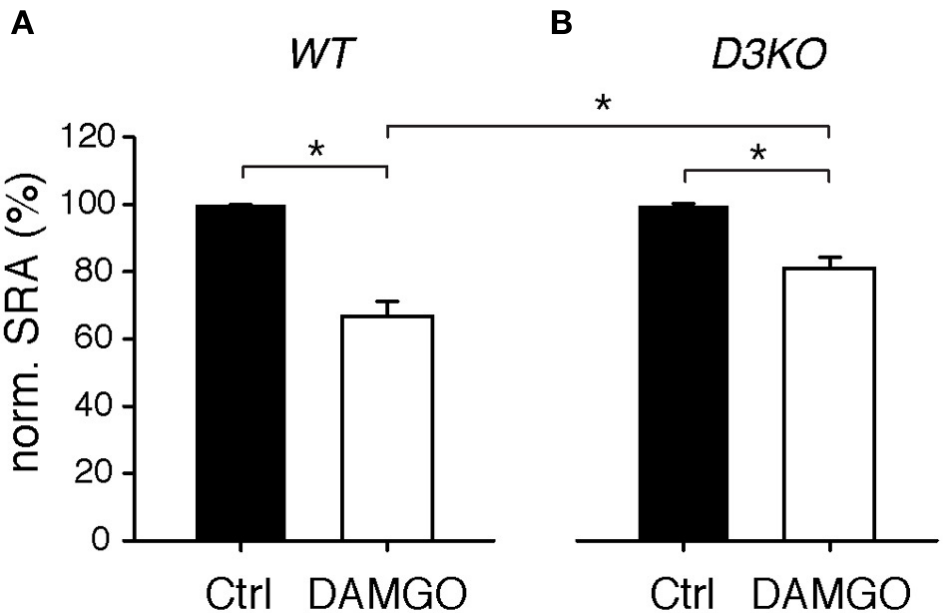
**Modulation of spinal reflex amplitudes (SRAs) by the MOR-preferring agonist, DAMGO. (A)** In WT, DAMGO induced a significant decrease in SRA (to 66.6 ± 4.5% of control, *n* = 15). WT: ^*^p < 0.001. **(B)** In D3KO, DAMGO equally significant reduced SRA, albeit to lesser degree than in WT (to 80.9 ± 3.4%, *n* = 8). D3KO: ^*^p < 0.001. The difference between DAMGO-induced effects in WT and D3KO was significant (*p* = 0.013).

While preferentially targeting MORs, morphine activates ∂ and κ receptors as well. To test if a targeted activation of the MOR pathway alone can mimic the effects observed with morphine, we bath-applied the MOR-preferring agonist, DAMGO (10 μM), to isolated WT and D3KO spinal cord preparations and assessed its role in modulating SRAs (Figure 4). In WT, DAMGO decreased SRAs from 99.9 ± 0.1 to 66.6 ± 4.5% (*n* = 15, *p* < 0.001, Figure 4A), and it decreased them in D3KO from 100 ± 0.15 to 80.9 ± 3.4% (*n* = 8, Figure 4B). Importantly, the difference in DAMGO-mediated actions between WT and D3KO was significant (*p* = 0.013). These data suggest that in isolated spinal cords of D3KO, MORs are still functionally active and can be recruited by a highly specific agonist, albeit at a smaller magnitude than in WT.

**Figure 5:**
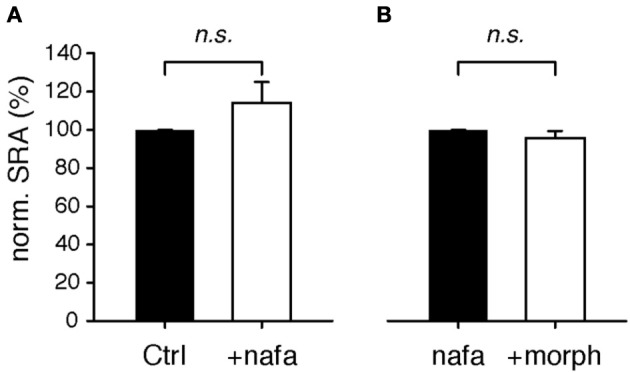
**Modulation of spinal reflex amplitudes (SRAs) by the D3-receptor preferring antagonist, nafadotride. (A)** In the isolated WT spinal cord, bath-application of nafadotride led to slight, albeit insignificant increase in SRAs (*n* = 8). **(B)** In the presence of nafadotride, additional application of 1 μM morphine failed to exert any modulatory action on SRAs (*n* = 5).

The D3KO animal is a functional, global knockout, and consequently D3 receptor function is compromised in every tissue. To test if D3 receptor dysfunction in the spinal cord is sufficient to mimic the morphine effects observed in the D3KO both *in vivo* and *in vitro*, we tested the consequence of an acute block of the D3 receptor system in the isolated spinal cord prior to morphine exposure (Figure 5). Following baseline recordings, WT spinal cords were exposed to the D3 receptor-preferring antagonist, nafadotride (Figure 5A), before additionally bath-applying morphine (Figure 5B). Application of nafadotride alone (20 μM) led to a slight, yet insignificant increase in SRA from 99.7 ± 0.2 to 113.9 ± 11.1% (*p* = 0.87, *n* = 8, Figure 5A). Subsequent application of morphine, in the presence of nafadotride, and at the same dose that caused the significant inhibition of SRAs under control conditions (cf. Figure 3) failed to exert any significant modulatory action (ctrl: 99.8 ± 0.1 vs. 95.5 ± 3.9%, *p* = 0.69, *n* = 5, Figure 5B). These data are testimony that an induced acute block of spinal D3 receptors is sufficient to prevent morphine effects.

### D1 receptor expression

**Figure 6:**
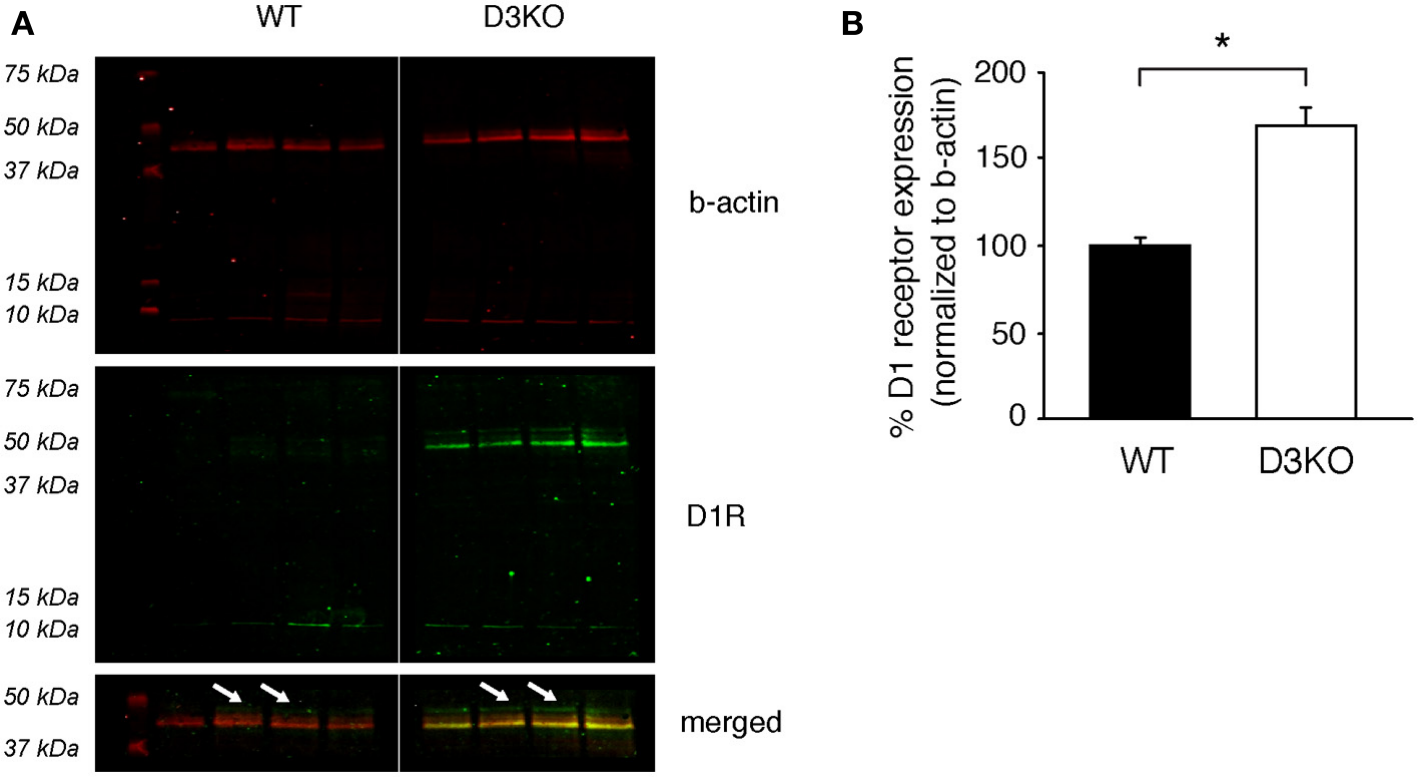
**Protein expression levels of the dopamine D1 receptor in lumbar spinal cord. (A)** ß-actin (top panels, red), D1R (middle panels, green), and merged images of the 37–50 kDa band of interest (bottom panels) of the same blot. Note that ß-actin expression is similar in WT and D3KO. In contrast, the D1R antibody recognizes a strong signal at the 42 kDa band that is present only in D3KO, and a weaker signal at the predicted band size of 49 kDa that is differently expressed in WT and D3KO (arrows) and that was used for the subsequent analysis. **(B)** The quantification of the 49 kDa expression labeling, normalized to ß-actin expression, revealed a significant increase in D1 receptor protein expression levels in D3KO (^*^p < 0.001).

As the D3 receptor is closely associated with the D1 receptor through heterodimerization (Surmeier et al., 1996; Fiorentini et al., 2008; Maggio et al., 2009; Missale et al., 2010; Cruz-Trujillo et al., 2013), we next tested if the dysfunction of the D3 receptor system leads to changes in the D1 receptor system (Figure 6). We found that in D3KO (*n* = 5), D1 receptor expression (49 kDa band) increased significantly from 99.99 ± 4.6% (in WT) to 170.6 ± 8.9% (*n* = 5, *p* < 0.001). These data support the notion that the increased excitability in the D3KO spinal cord could be, at least in part, driven by an increased expression in D1 receptor levels.

## Discussion

We show here that a dysfunction of the dopamine D3 receptor is associated with a morphine-resistant state *in vivo*, that this behavioral phenotype can be mimicked in the spinal cord *in vitro*, and that this altered morphine responsiveness is associated with an increase of pMOR but not total MOR expression in the lumbar spinal cord. In addition, we found that an acute block of D3 receptor function in the isolated spinal cord completely abolished the modulatory capabilities of morphine on SRAs, suggesting that the disruption of D3 receptor function *in vitro* is sufficient to induce a state of morphine resistance.

The D3 receptor is part of the D2-like family, and an evaluation of D1- and D2 receptor agonists, at a time when other DA receptors had not yet been described, reported that D2 activation produced antinociception, whereas D1 receptor activation induced mild hyperalgesia (Rooney and Sewell, 1989). More recently it was shown that co-administration of morphine with nafadotride could effectively suppress the morphine-induced behavioral sensitization observed in WT mice after acute morphine administration (Li et al., 2010), and it was suggested that D3 receptors regulate basal nociception (Li et al., 2012). Together, these data support our findings that the D3 receptor may have a critical role in regulating the morphine response in the spinal cord.

Loss of morphine analgesia is generally associated with a reduction in functional MOR receptors, either through down-regulation or desensitization of receptors (Williams et al., 2013). Such a down-regulation is characterized by a decrease in functional receptors present on the cell membrane due to degradation or decreased biosynthesis (Finn and Whistler, 2001). We found that D3KO and WT mice had similar levels of total MOR expression indicating that MOR receptor down-regulation *per se* is not the primary mechanism behind the lack of morphine responsiveness in D3KO mice *in vivo* (Figure 2). Our studies, however, were insensitive to receptor distribution between cellular compartments which would effect of the availability of functional MOR. Desensitization involves molecular changes at the receptor signaling level (Yu et al., 1997; Bohn et al., 2000), with receptor phosphorylation being the first step. MOR desensitization is under the control of GRK-mediated phosphorylation (Connor et al., 2004; Gainetdinov et al., 2004; Garzon et al., 2005), followed by ß-arrestin-mediated internalization (Bohn et al., 1999, 2000; Ohsawa et al., 2003; Connor et al., 2004). Our data show that, while total MOR protein levels were similar across groups, a greater proportion of those receptors were phosphorylated in D3KO compared to WT, suggesting that these receptors were desensitized and will not effectively signal, even in the presence of a ligand (Figure 2). While the MOR can be phosphorylated by both GPCR kinase (GRK) and non-GRK mechanisms, the antibody used for this experiment was designed to recognize phosphorylation of the Ser375 residue of the MOR, a site that is critical for GRK phosphorylation, arrestin recruitment and endocytosis (El Kouhen et al., 2001). This site has also been shown to be phosphorylated in states of morphine tolerance and with sustained release of β-endorphin, the endogenous ligand for MOR (Petraschka et al., 2007). Such alterations in binding efficiency of MOR to endogenous opioid ligands may provide one explanation for our findings why D3KO mice have lower baseline thermal pain thresholds than WT (Keeler et al., 2012), and they may also explain the lack of analgesia with exogenous morphine treatment. Studies assessing GRK activity levels and endogenous opioid levels in D3KO mice are needed to determine if the baseline phosphorylation of the MOR results from either increased GRK activity and/or continuous activation due to an increased availability of endogenous ligand.

A possible limitation of our immunohistochemical findings is that the commercially-generated antibodies used in our study were not, or only to a limited extent, validated in external peer review processes, and thus may not be optimized or only partially effective in detecting the proteins of interest (e.g., the 42 kDa band we observed exclusively in the D3KO spinal cord when probing for the D1 receptor, Figure 6). However, as the 49 kDa band (s. arrows in Figure 6A) corresponds to the predicted D1 receptor protein size, has together with a 75 kDa band also been observed at this size recently (Mizuta et al., 2013), and was both present and differentially expressed in WT and D3KO (Figure 6B), we are confident that future work will corroborate the molecular changes reported in this study. At the same time, given the high structural homology between D1 and D5 receptors, we can not exclude the possibility that such studies may identify an up-regulation of the D5 receptor instead of the D1 receptor as suggested by our findings. Yet, given the similar functional properties of D1 and D5 receptors, both of which primarily mediate excitatory actions, and the lack of evidence of D1/D5 receptor interactions, we have based our model on the existing literature that has identified D1/D3 interaction and heterodimers, which could act in synergistic fashion to control and mediate DA and morphine actions.

Our finding that the MOR agonist DAMGO can induce a modulatory response in both WT and D3KO could be the result of its higher specificity in activating the MOR than morphine (Minami et al., 1995; Onogi et al., 1995; Saidak et al., 2006), which also activate ∂-receptors (Walwyn et al., 2009), and/or its high affinity to the MOR (Pak et al., 1996). As we observed lower levels of non-p-MOR in D3KO than in WT (Figure 2), it is conceivable that DAMGO but not morphine may be able to activate this receptor population in D3KO, thus allowing the modulation of the SRAs *in vitro*.

**Figure 7:**
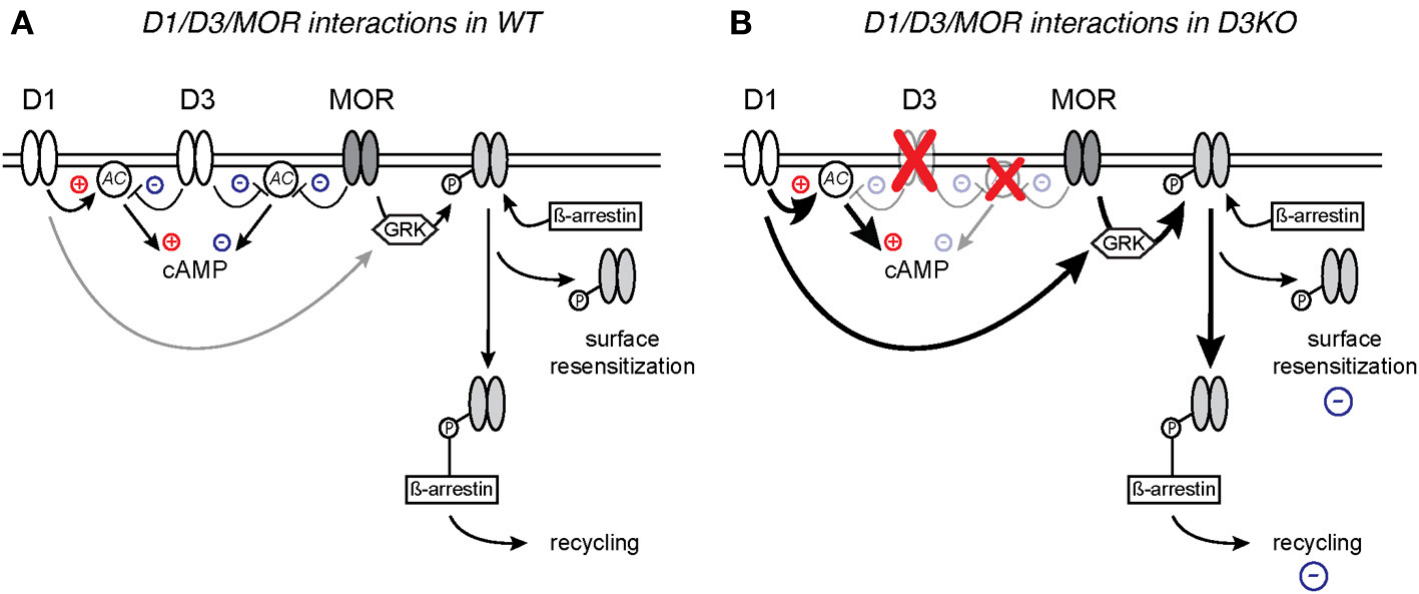
**Proposed model of D1 and D3 receptor interactions with MOR in WT (A) and D3KO (B). (A)** Naïve WT with intact D1, D3, and MOR signaling pathways. **(B)** D3KO with compromised D3 function and altered second messenger cascades.

Resistance to opioid treatment involves in part the DA system, including the D3 receptor system (Richtand et al., 2001; Sokoloff et al., 2001; Kosten et al., 2002; Vorel et al., 2002; Le Foll et al., 2007; Heidbreder, 2008; Hell, 2009; Li et al., 2012). Such tolerance can arise from a dysfunction of MOR and DA receptor mediated cAMP/PKA second messenger pathways in the brain (Zhao et al., 2007; Barraud et al., 2010). Both DA and MORs are located pre- and post-synaptically in spinal cord sensory neurons (Xie et al., 1998; Levant and McCarson, 2001; Abbadie et al., 2002; Millan, 2002) and they control the activation of adenylyl-cyclase (AC). Generally, DA D1-like receptors (D1, D5) mediate excitatory actions by increasing AC activation and raising cAMP levels in the target neuron (Missale et al., 1998; Neve et al., 2004), whereas D2-like (D2, D3, and D4) and MOR pathways reduce AC activation and decrease cAMP levels (Yu et al., 1990; Jaber et al., 1996; Mamiya et al., 2001; Neve et al., 2004; Sheng et al., 2009). As D1 and D3 receptors often co-localize or form heterodimers (Karasinska et al., 2005; Fiorentini et al., 2008, 2010; Marcellino et al., 2008; Beaulieu and Gainetdinov, 2011) and oppositely regulate cAMP/PKA-mediated second messenger pathways, it is conceivable that the dysfunction of the D3 receptor might directly modify D1 receptor function. While we previously did not observe differences in D1 mRNA expression in the D3KO spinal cord (Zhu et al., 2008), probing for the affiliated protein expression revealed a significantly increased D1 receptor protein expression in D3KO (Figure 6), supporting the notion of a D3-mediated influence on D1 function. Additionally, the similarities in MOR- and D3-mediated second messenger pathways suggest that they may have synergistic effects on reducing pain transmission (Li et al., 2012; Saghaei et al., 2012), and the co-localization of D1 with D3 receptors suggest that interactions in their common cAMP/PKA signaling cascade might play a role in the emergence of the morphine resistance seen in D3KO (Figure 7).

In WT, a nociceptive stimulus induces the release of DA and endogenous opioids, including beta-endorphin, which preferentially bind the D3 and MOR, respectively, and initiate signaling cascades that inhibit cAMP production, resulting in the opening of K ^+^ channels or closing of Ca^2+^ channels (Connor and Christie, 1999; Connor et al., 1999; Williams et al., 2001) thus decreasing the transmission of the nociceptive signal. MOR binding is followed by GRK-mediated phosphorylation (Connor et al., 2004; Gainetdinov et al., 2004; Garzon et al., 2005), and ß-arrestin-mediated internalization (Bohn et al., 1999, 2000; Ohsawa et al., 2003; Connor et al., 2004) of the receptor, followed by recycling or de-phosphorylation and subsequent reinsertion into the membrane. More recent evidence also demonstrates that recovery from phosphorylation and desensitization can occur on the cell membrane, without the need for endocytosis of the receptor (Arttamangkul et al., 2006; Doll et al., 2011).

This intracellular overlap between the MOR and DA systems, along with evidence that D1, but not D2 receptors are involved in mediating the behavioral responses to morphine by acting to recruit GRK and ß-arrestin to the MOR (Urs et al., 2011) and that blocking ß-arrestin expression has been shown to enhance morphine-mediated analgesia (Li et al., 2009), has led to the model proposed in Figure 7. According to our model, activation of the D3 receptor leads in WT to a reduction of adenylate cyclase (AC) activity, which will result in a reduction of cAMP levels and decreased cAMP-mediated signaling, in a manner that is synergistic to the MOR in response to its ligand. However, under this scenario, D1 activation can also activate AC and compensate for a D3- or MOR-mediated reduction in cAMP levels, thus permitting a bi-directional modulation of cAMP-mediated pathways. As D1 and D3 receptors often co-localize or form heterodimers in the brain (Karasinska et al., 2005; Fiorentini et al., 2008, 2010; Marcellino et al., 2008; Beaulieu and Gainetdinov, 2011) and oppositely regulate cAMP/PKA-mediated second messenger pathways, we postulate that, in D3KO, the dysfunction of the D3 receptor prevents the D3-mediated block of AC, and leaves D1 receptor actions unopposed. Under such circumstances, cAMP pathways might be continuously up-regulated, and additional application of cAMP nucleotides might fail to further increase cellular excitability. Further, activation of the D1 receptor induces ß-arrestin signaling complex formation, in which β-arrestin acts as a scaffold for different kinases and phosphatases (Beaulieu et al., 2005; Urs et al., 2011) which in turn may lead to desensitization of receptors through G protein-independent signaling mechanisms (Pierce and Lefkowitz, 2001; Lefkowitz and Shenoy, 2005) and increased pMOR levels. We postulate that these changes to the second messenger systems, either individually or in concert, can create the overly excitable cellular state that is evidenced by decreased sensory thresholds at baseline, and the resistance to the analgesic effects of the opiates.

Taken together, our data reinforce the idea that changes in the ability of opioids to provide analgesia can arise from a dysfunctional D3 receptor, as demonstrated using spinally-mediated behaviors and reflex circuits, and that this unresponsiveness to morphine can be induced acutely in the isolated spinal cord by blocking the D3 receptor. Therefore, the D3KO mouse may be a powerful tool with which to study the alterations to the MOR second messenger-signaling cascade, to decipher the initial mechanisms that may underlie the waning of morphine effectiveness over time.

### Conflict of interest statement

The authors declare that the research was conducted in the absence of any commercial or financial relationships that could be construed as a potential conflict of interest.
